# Professionals’ views and experiences of the TrainDEEP (TRaining Assistance INitiative in DEep End Practices) pilot: transforming GP practices into training practices in disadvantaged areas in the North East of England

**DOI:** 10.3399/BJGPO.2025.0090

**Published:** 2026-05-06

**Authors:** Alisha Gupta, Mihirini Sirisena, Gillian Vance, Matthew Armstrong, Sarah Sowden

**Affiliations:** 1 Population Health Sciences Institute, Faculty of Medical Sciences, Newcastle University, Newcastle-upon-Tyne, UK; 2 School of Medical Education, Newcastle University, Newcastle-upon-Tyne, UK

**Keywords:** inequalities, health inequities, community care, general practice, qualitative research

## Abstract

**Background:**

The North East and North Cumbria Deep End (NENC DE) Network comprises GPs and primary care staff serving the most socioeconomically disadvantaged communities. GPs often work in practices where they have trained, yet there are fewer training practices (TPs) in the Deep End (DE), limiting recruitment opportunities. TRaining Assistance INitiative in DEep End Practices (TrainDEEP) aims to increase TP numbers, to enhance GP recruitment, and to improve healthcare access for communities with the greatest need.

**Aim:**

This study aimed to explore the views and experiences of professionals involved in the TrainDEEP intervention designed to transform DE practices into TPs to enhance GP recruitment and retention in socioeconomically disadvantaged areas.

**Design & setting:**

A qualitative longitudinal study involving professionals working in two DE practices in the North East of England, who were part of the TrainDEEP intervention.

**Method:**

Semi-structured interviews were conducted at two points during the 12-month intervention. Transcripts were coded inductively and thematically analysed.

**Results:**

A total of 17 interviews with 10 participants were conducted (September 2023 to February 2025). The following three main themes emerged: (1) becoming a TP, including resource provision and barriers; (2) training in a DE context, focusing on learning opportunities, and recruitment and retention challenges and solutions; and (3) impact on the practice, evaluating TrainDEEP's success and future sustainability..

**Conclusion:**

The TrainDEEP model was successfully implemented, receiving positive feedback. This model could be expanded across the local and national DE networks to increase TPs, boosting recruitment and enhancing healthcare provision for underserved communities.

## How this fits in

Patients in socioeconomically disadvantaged areas have complex biopsychosocial needs and greater health demands than those in affluent areas. Socioeconomically disadvantaged areas also face higher rates of staff burnout and lower GP recruitment and retention. Research shows there are fewer training practices in socioeconomically disadvantaged areas, where GPs see more patients per doctor. This study evaluated an intervention designed to increase training practice numbers and enhance recruitment and retention in socioeconomically disadvantaged areas.

## Introduction

The inverse care law states that there is an inverse relationship between the availability of high-quality medical care and the demand for it in the population, meaning that those with greater health needs are less likely to receive timely care.^1^ GPs are unequally distributed in the UK, with fewer in socioeconomically disadvantaged areas (SDAs).^2^ Practices in SDAs struggle to recruit and retain doctors,^3^ leaving GPs in SDAs responsible for 300 more patients per doctor annually than those working in affluent areas.^4^ This is owing to several reasons, including managing patients with multiple comorbidities, complex psychosocial needs, and demanding workloads, all contributing to high stress and burnout levels.^5–8^

There are fewer training opportunities in SDAs owing to the lower number of training practices (TPs), limiting GP registrars' exposure to working in these settings and the additional challenges posed by this, such as having many patients with complex biopsychosocial presentations and conducting a high proportion of consultations using intepreters.^9,10^ Studies show that registrars tend to stay in areas where they trained,^5^ so being a TP is important for recruitment and retention.

The Deep End Network (DEN) was established in Scotland in 2009, and the North East and North Cumbria (NENC) DEN was established in 2020 to support professionals and the communities they serve that experience extreme socioeconomic disadvantage. There are 52 Deep End (DE) practices in the NENC DEN, where half or more of their patients live in the 15% most socioeconomically disadvantaged neighbourhoods in the country, according to the Indices of Multiple Deprivation (IMD) 2019.^11^ In August 2023, only 42% (*n* = 22) of NENC DEN practices were TPs, compared with 70% (*n* = 244) across all general practices in the NENC region.

To support recruitment and retention, and improve healthcare access, the NENC DEN^12^ and a local GP training programme director launched the TrainDEEP pilot, funded by the NHS NENC Integrated Care Board (ICB). An experienced trainer supported DE practices in becoming TPs, with the intervention delivered in two phases.

### Phase 1 (months 0–6)

An experienced GP trainer visited the DE practice twice weekly to upskill the new trainer and support the transition into a TP. They also provided clinical cover, giving the DE GP protected time to complete an intending trainer’s course.

### Phase 2 (months 6–12)

A GP registrar joined the practice, receiving joint supervision from the experienced and new trainer. The experienced GP trainer continued to deliver weekly clinical sessions.

The 12 months were expected to be sufficient for meeting training requirements, after which the experienced trainer would move to another practice to replicate the model.

The registrar joined the practice in either February or August, in line with the national postgraduate GP training rotation dates. In this way, the TrainDEEP initiative is fully compatible and integrated with the usual postgraduate training pathway.

On completion of the scheme, the DE practices become accredited TPs and are then able to host registrars, supervised by the new GP trainer.

This study aimed to qualitatively explore the benefits, challenges, and feasibility of transforming DE practices into TPs through the TrainDEEP intervention to enhance GP recruitment and retention in SDAs.

## Method

A qualitative longitudinal semi-structured interview^13^ study was undertaken. 

Practices were recruited into the intervention as follows: before the intervention (August 2023), 30/52 (58%) of DE practices were not TPs and were therefore eligible to participate in the TrainDEEP intervention.

Two practices self-selected to participate through an open expression of interest call, which was emailed out across the NENC DEN member practices. Any DE practice that was not currently a TP could apply. The experienced GP trainers were recruited through word of mouth at DE and GP educational networking events. The GP registrars were allocated to the practices as part of their standard training programme.^14^

Participants were recruited for the qualitative research project via convenience sampling from the two DE practices in the NENC DEN that were taking part in the TrainDEEP intervention.

An invitation email was sent to professionals working in the pilot practices, with an attached participant information document (Appendix 1) and consent form (Appendix 2). Specific clinical and non-clinical professional roles across the two practices were purposively recruited owing to their involvement in the practice and the insights this afforded them about the intervention. Interviews were requested at repeat points in time over the lifecycle of the intervention.

### Data collection

Participants completed electronic consent forms. One-to-one semi-structured interviews lasting 20–45 minutes were conducted via Microsoft Teams (version 1.6.00.24078). These were based on topic guides (Appendices 3a–b) and were completed in both phases of the pilot. Interviews were conducted by AG, an academic foundation doctor, and MS, a research associate, between September 2023 and February 2025.

### Data analysis

Interviews were transcribed by an external transcription company and checked for accuracy and errors by AG. Qualitative data analysis was conducted using a grounded theory approach, following Braun and Clarke’s six-stage thematic analysis framework.^15^ AG read the transcripts for familiarisation and data immersion before using an inductive approach^16^ to develop codes. NVivo (version 14) was used for coding and organising codes into broader themes to highlight patterns across the data. AG and MS discussed codes and themes to improve the rigour of interpretation. Following this, themes were refined and finalised.^17^

## Results

### Participants

There were 10 participants, seven were interviewed twice, and three were interviewed once, totalling 17 interviews. For both practices, interviews were done with the experienced GP trainer, the intending trainer, the practice manager, a second GP partner, and the registrar.

The practice characteristics are outlined in Table 1.

**Table 1.** Practice characteristics

Practice	A	B
List size^26^	7819	8237
Number of GPs	5	5
Location	Urban coastal	Inner city
Mean patient age, years^27^	47.61	33.33
Patient gender, Male: Female %^27^	Male: 43.5% Female: 56.4%	Male: 59.8% Female: 40.2%

### Themes

The analysis identified the following three key themes, with several sub-themes outlined in the thematic map (Figure 1):

1. Becoming a TP
2. Training in a DE context
3. Impact on the practice

**Figure 1.**
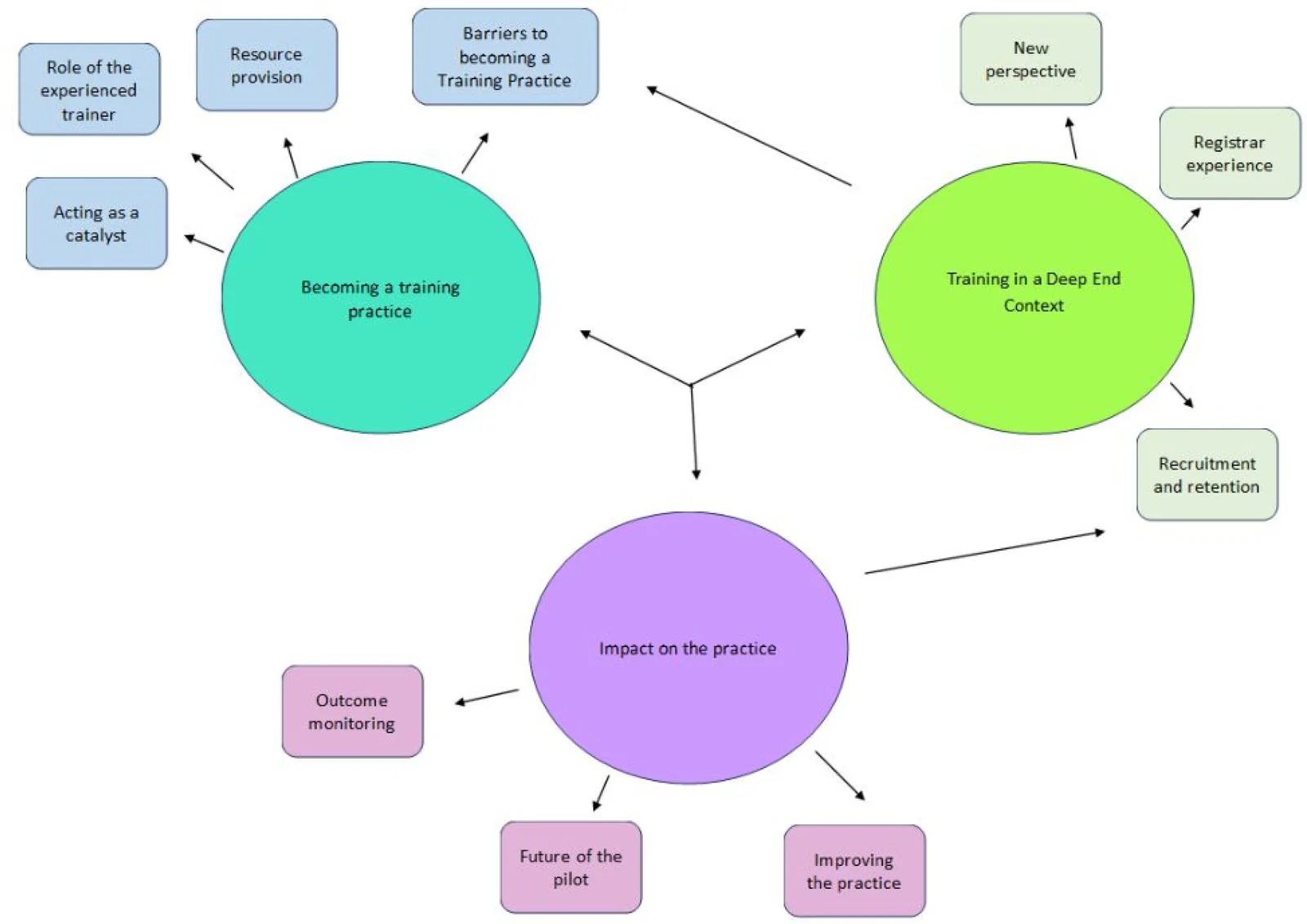
Thematic analysis map demonstrating themes and sub-themes

#### Becoming a TP

Participants reported barriers such as limited time to meet TP requirements and attend the trainer’s course. TrainDEEP addressed these by providing an experienced trainer, which is the key to its success. This support helped practices achieve TP status sooner than they would have otherwise.

##### Barriers to becoming a TP, acting as a catalyst, resource provision, and role of the experienced trainer

Both practices were keen to become a TP, but mentioned several barriers that delayed this ambition. Participants outlined that there were requirements for the intending trainer’s course, such as recording consultations, which is time-consuming and requires setup, patient explanations, and consent. Completing these tasks, on top of a heavy workload, is challenging, and it was mentioned that clinicians felt like they lacked the capacity to meet the requirements for the course:

*‘To go on the course, you have to submit videos with your consultations, and they’re assessed. That’s a barrier. I think going back to videoing your consultations is a bit daunting.’* (Intending trainer, Practice A)

Participants attributed the pilot’s success to the experienced trainer, who used their expertise to guide the practice in meeting training requirements, ensuring early compliance and avoiding trial-and-error. Their clinical support also gave the new trainer protected time for their course, allowing the practice to become a TP sooner than without TrainDEEP’s help:

*‘The pilot gives the gift of resource rather than the gift of money, there’s an experienced trainer, an experienced clinician, who can provide clinical support to the practice by seeing patients.’* (Experienced trainer, Practice A)

### Training in a Deep End context

DE practices struggle with recruitment and retention, so participants hope that becoming a TP will prepare registrars to work in an SDA, support their development, and encourage them to return after qualifying. Training status also brings teaching and leadership opportunities, making it attractive to qualified GPs.

#### Recruitment and retention

To combat recruitment and retention issues, participants believed that becoming a TP would increase the likelihood of attracting recently qualified GPs. They referred to the fact that it is known that GPs often settle where they train, so hoped that positive DE training experiences will encourage registrars to return post-qualification:

*‘I was a trainee at the practice where I was a partner for 15 years. There is evidence to say that if you are a trainee in a practice, there is a higher chance that you will be recruited there.’* (Experienced trainer, Practice A)

Additionally, participants felt that being a TP raises awareness of the DE by word of mouth and creates a network of potential GPs to recruit, as registrars would share their experiences with their peers. They hope that this could improve recruitment rates in the future:

*‘Training practices seem to be able to recruit from ex-trainees or trainees that know trainees, if you’re not a training practice, it’s hard to attract in.’* (Intending trainer, Practice A)

Participants outlined that being a TP boosts a practice’s quality and reputation, which could attract doctors by offering education and training opportunities, enabling them to develop additional skills. They hoped that by providing registrars with meaningful learning experiences, it would increase the likelihood of their return:

*‘Being a training practice drives up the quality of the practice. It makes it more desirable to work in. If you’ve got trainees who have good experiences, they’re more likely to come back, as fully qualified GPs, to work there. It’s to try to help recruitment and retention in those practices.’* (Intending trainer, Practice B)

Participants explained how they struggle to retain staff owing to high workloads and patients with complex needs. They believe that training registrars will equip them with the skills and experience to manage the challenges of working in the DE. Participants hoped a registrar would help alleviate the clinical workload, thereby reducing pressure on staff and locum usage, which could lead to lower burnout rates and improved retention in the future:

*‘Because of the kind of practice, we are, there were huge problems with recruitment and retention. Many people were coming to work here six months and then leave because it is a difficult set of patients that we look after.’* (GP partner, Practice B)

### Registrar experience

Participants noted that patients in the DE present with complex biopsychosocial issues and earlier-onset comorbidities, offering valuable learning for registrars. Exposure to uncommon health problems helps develop consultation skills, benefiting both trainees and future patients. Staff described the work as meaningful and hoped it would inspire registrars to pursue careers in DE practices:

*‘It’s really good for the trainee. It’s a great training post to have so many tricky things. Learning to consult with interpreters, people whose second language isn’t English, people whose health literacy is poor. If you can do that here, you can go anywhere. You make a lot more diagnoses here. You pick up a lot more things. I’ve diagnosed things here that I’ve never diagnosed in my full career, like hepatitis B, TB* [tuberculosis] *, and infectious diseases. Higher rates of cancers, two-week wait referrals, serious mental illness. It’s not great for the patients, but from a training point of view it’s really good.’* (Experienced trainer, Practice B)

Registrars felt that they gained additional experience and confidence in an SDA owing to accommodating practices. Placements were structured to allow shadowing of the multidisciplinary team before independent patient care, thereby fostering a supportive learning environment:

*‘If I feel I'm not getting enough exposure to certain things, it’s been easy to address. Like safeguarding, something I had not much exposure to, so chatted to supervisors and they freed time so I could attend safeguarding meetings. My supervisors and the practice were very supportive.’* (GP registrar, Practice B)

Registrars described how they would be keen to return to work in the DE practice following completion of their training, due to positive experience of their placement there:

*'If the opportunity arose, I'd be very happy to go back to* [Practice A] *at the end of my training if there was a space and they were happy to have me, because I've learned that it's a lovely practice and somewhere that I'd be happy to work.'* (GP registrar, Practice A)

#### New perspective

Participants acknowledged that registrars increase trainer workload through teaching, but their contribution to clinical patient care balances this. They felt that trainees also bring current knowledge and secondary care experience, offering valuable insights and suggesting improvements for future training placements:

*‘You have extra clinics, so it’s like a free clinic. But you can also learn from them. They come in with fresh ideas, fresh eyes. It’s a good experience for the patients, it’s a good experience for the staff, it’s good support for the clinical team.’* (Practice manager, Practice B)

### Impact on the practice

TrainDEEP’s success can be monitored in several ways, with participants highlighting the positive impact on staff morale. They emphasised that future success depends on the experienced trainer’s ability to adapt quickly, enabling the intending trainer to complete their course.

#### Outcome monitoring

Staff discussed several ways to measure outcomes, citing examples of short-, mid-, and long-term impacts.

Participants explained that they have been considering becoming a TP for some time, as they were aware of the benefits it could bring to both staff and patients; however, owing to the barriers aforementioned, they have not been able to. This is why they reported that a short-term measure of success was simply receiving the TP accreditation:

‘Su*ccess is that we become a training practice*.’ (Practice manager, Practice A)

To measure mid-term success, suggestions included capturing registrar feedback to highlight strengths and areas for improvement. It was felt this would demonstrate that the practice has been accommodating, and had given the registrar opportunities to learn and develop their clinical and professional skills in line with their curriculum. Participants mentioned that they could use this feedback to reflect and make changes to placements in accordance with this feedback:

*‘The best metric is training success. Do we always have a trainee, and do they provide good feedback? Are there successful GP placements with happy trainees?’* (Intending trainer, Practice B)

Participants discussed how longer-term success could be measured by monitoring recruitment and retention over time, focusing on the number of registrars who have had placements in DE practices and subsequently return to the practice or area once qualified. GP retention rates could be measured and compared with pre-TP levels to assess the impact of becoming a TP. Some felt that this could also reduce the number of locums, thereby improving the continuity of care and potentially enhancing the health outcomes of patients:

*‘... if you can recruit and retain doctors, you can staff a practice better, you can invest in your patient better which leads to better patient and population health outcomes.’* (Experienced trainer, Practice A)

In addition to registrar feedback, patient feedback was also reported as an important component of measuring success. Practice staff felt that this could be measured by looking at internal data and collecting feedback from patients surrounding their experiences with accessing services. It was suggested that routine data on patient appointment waiting times could be monitored to identify the impact of additional clinical capacity on waiting times.

*‘It’s a positive for the patients that we have more appointments to offer.’* (Practice manager, Practice A)

#### Improving the practice

Staff were excited to become a TP, and having a registrar in the practice has improved staff outlooks. With staff members feeling burnt out and drained, TrainDEEP has brought positive changes to the practices:

*‘It’s nice for the patients and the staff as well. It’s a good morale thing. This is positive for the practice. This will enable us to stay as the practice we are.’* (Practice manager, Practice B)

TrainDEEP was a new initiative for the practices to develop, and participants felt that this could create new opportunities moving forward, such as helping to upskill other practices. There is also potential for practices to share information about recruitment opportunities within the DEN:

*‘We can look at supporting other Deep End practices with signposting trainees that we’ve had that are looking for jobs to them or, if they’re looking at becoming a training practice, if we can support them as well*.’ (Intending trainer, Practice A)

#### Future of the pilot

When planning for the future of the pilot, several considerations were discussed. Staff acknowledged that patient numbers have been increasing over time, and there are growing numbers of allied health professionals in practices. Practices lack the physical space to host a registrar, so it was highlighted that they will need to expand their capacity to take on multiple registrars in the future, once they become a more experienced TP:

*‘If the practice was to take on another trainee, they might find it challenging because, unless there’s significant investment in the estate to create rooms, it’s going to cause challenges with regards to being able to expand and have space for more trainees.’* (Experienced trainer, Practice B)

Participants felt that a crucial component for the success of the intervention was the provision of an experienced trainer. They outlined key traits the trainer should have for TrainDEEP to be successful: that they are an experienced clinician, and have an appreciation for working in the DE and the additional challenges posed by this work. They underlined how the trainer will have to quickly adapt as they will be covering clinical sessions soon after joining the practice, so there needs to be a smooth transition to enable the intending trainer to complete the course, knowing that their clinical work is being appropriately covered:

*‘There are certain attributes the experienced trainer has to have to be able to undertake TrainDEEP. They have to be experienced, enthusiastic. They have to be willing to work in and understand what it means to work in a Deep End practice.’* (Experienced trainer, Practice A)

## Discussion

### Summary

Those working in the DE felt the TrainDEEP model had successfully streamlined the transition of their practices into TPs. Participating in the intervention was a rewarding experience for those involved, lifting staff morale. Participants found the experienced trainer’s knowledge and expertise invaluable, ensuring they met all the requirements to become a TP on their first attempt. They acknowledged that the clinical cover from the experienced trainer alleviated several barriers to becoming a TP, including having enough time to meet the demands of the intending trainer’s course alongside high workloads.

DE practices struggle to recruit and retain doctors, and it is hoped that being a TP will help with this recruitment and retention, as it provides a steady stream of registrars who may return once qualified, reducing locum use and improving care continuity. The work is challenging and offers unique learning opportunities for registrars.

Professionals generated ideas around monitoring the impact of TrainDEEP, highlighting important short-, medium-, and long-term outcomes. Staff discussed ways to measure the success of TrainDEEP, including accreditation as a TP and taking on trainees, and positive feedback from registrars and patients. In the long term, success could be measured by improved recruitment and retention rates and, ultimately, improved patient care.

In the future, practices that have undertaken TrainDEEP could take on multiple registrars; however, many practices lack physical space to host registrars and need funding to expand. As mentioned in our findings, the experienced trainer is a linchpin for the intervention, but there are a limited number of experienced trainers with these specialised skills and capacity.

### Strengths and limitations

To our knowledge, this is the first research exploration of the use of a model embedding an experienced GP trainer into a DE practice to transform it into a TP.

Qualitative semi-structured interviews with a range of staff members provided diverse insights, enhancing data richness. Longitudinal interviews allowed for the exploration of evolving perceptions throughout the pilot. Two practices were involved, enabling comparisons across different geographical areas, patient populations, and organisational structures.

Both researchers were familiar with the DE area of work and research, enabling in-depth discussions with interviewees during data collection. Two researchers worked on the data analysis, and codes and themes were discussed to improve the richness of interpretation and to enable theme refinement. The wider research team were involved in discussions on theme development, strengthening the robustness of the analysis.

Regarding the limitations, this small qualitative study in two self-selecting NENC practices has limited generalisability; experiences reported may not be consistent with findings in other practices if the intervention were to be rolled out across a broader footprint. In future, factors affecting the generalisability of the findings could be captured, such as the demographic and socioeconomic characteristics of the participants. As an early pilot, mid-to-long-term success and potential issues (for example, poorly performing trainees,^8^ burnout rates) are yet to be fully measured and understood.

The study only included interviews with professionals involved in the pilot. It would have been valuable to include the views of local GP training educators, particularly for their guidance on defining success and capturing future outcomes. This would add valuable context to the study, as their knowledge of GP practices in the area would help determine the degree of generalisability to other NENC DE practices.

The practices involved in the intervention and research were recruited by convenience sampling, which can lead to selection bias as practices that wanted to participate, or had expressed an interest, were given the opportunity, and others who were unable or did not want to participate missed out. A limitation of the research, therefore, is that it does not explore the views of those who did not express an interest in participating in the intervention and it would have been interesting and insightful to have understood the barriers they face. It is possible that even with comprehensive support offered through TrainDEEP, there may not be an appetite in other practices for becoming TPs and it would be important to explore this possibility.

### Comparison with existing literature

Previous literature highlights that there is a lower distribution of TPs in SDAs compared with affluent areas.^10^ Unlike other UK^18^ and Ireland^19^ GP programmes targeting existing TPs, TrainDEEP focuses on developing new ones in disadvantaged areas. This study shows that the TrainDEEP model has been effective in both practices.

Current literature shows that recruitment and retention in general practice is declining,^3^ particularly in DE areas,^5^ and TrainDEEP offers a potential solution to improve this decline. Workload has been cited as the main reason for not becoming a TP,^8^ and our findings align with this barrier. This research delves into additional barriers, in line with previous research,^8^ such as lacking the time and knowledge to become a TP, which the TrainDEEP model alleviates through the provision of an experienced trainer. The benefits also chime with existing research, notably improving morale,^8^ and add that being a TP could make it more attractive when recruiting, as it offers teaching and leadership opportunities.

Existing work shows that GPs are more likely to return to practices where they have trained, so this model was developed to increase the number of TPs in DE areas to improve recruitment and retention.^8^ Our findings highlight that by becoming TPs, practices are able to host registrars who are in turn exposed to working in an SDA, improving their confidence and potentially increasing the likelihood of them returning in the future. Registrars report that a supportive practice enhances their experience,^20^ and TrainDEEP participants felt well supported and would consider returning in the future.

Previous data also show that training in an SDA equips registrars with skills to work in a wide range of settings,^21^ and this was felt among TrainDEEP participants also, who felt that training in a DE practice exposes them to different challenges compared with a practice in an affluent area.

Recent data show a decline in overall GP numbers but a rise in registrars, coupled with increased GP unemployment. This is linked to stress and workload, causing many qualified GPs to work less than full-time.^22^ In our interviews, burnout and high workloads were cited as barriers to recruitment and retention. Work conducted in Northern Ireland shows that current TP distribution does not reflect the general population.^23^ Local data demonstrate that this is also apparent in the NENC region, and TrainDEEP aims to boost GP representation in SDAs, providing new opportunities for prospective GPs.

Research has been conducted to investigate the experiences of working in a DE practice,^24^ and this has been furthered in our research, concluding that despite the challenging work, training in the DE is highly rewarding and provides registrars with a range of learning opportunities to make them well-rounded doctors.

### Implications for research and practice

This research highlights that the TrainDEEP model was well-implemented and well-received in both practices. Participants suggested ways to monitor the impact and success of TrainDEEP, some requiring long-term evaluation. Practices could collate and evaluate feedback after multiple registrars have trained at the practice. Registrars could also be followed up throughout their training to monitor how their experience of working in a DE practice has impacted their training.

There is limited evidence surrounding the correlation between the level of deprivation and TP status, particularly in England. Research including practices in Scotland has been done, showing that the link between TP status and level of deprivation decreases as the practice list size increases;^10^ however, further work is needed.

Expanding the TrainDEEP model to other practices in the NENC region and beyond would provide a broader representation of the UK population, improving the external validity of the project. Further research is required to develop an understanding of how the current climate and challenges in general practice^25^ will impact the ongoing support, sustainability, and scale-up of TrainDEEP. This will help to ensure that a practice remains a TP and can be offered personalised support if required. The NENC DEN is also working to increase awareness and improve experienced trainer recruitment for TrainDEEP through engagement events, to allow more practices to become TPs.

There was an expression of interest form that went out to DE practices, but no follow-up with practices that did not engage with the intervention. This is an area for further research as it is important to understand and alleviate barriers in practices that do not express an interest in participating.

DE practices want to become TPs but face many barriers. The study highlights that the TrainDEEP model, particularly the provision of the experienced GP trainer, has been a model to create TPs and reduce the barriers faced by DE practices. This study can inform policymakers on a model to increase training opportunities and improve recruitment and retention of GPs in SDAs to help tackle the inverse care law.

## Data

The data supporting the findings of this research are available from the corresponding author on reasonable request.
